# A framework for reproducibly managing coupled research software and data assets based on shared transformation functions

**DOI:** 10.1016/j.patter.2026.101493

**Published:** 2026-04-13

**Authors:** Patrick Kuckertz, Benjamin Fuchs, Julian Schönau, Hedda Gardian, Kevin Knosala, Eugenio Salvador Arellano Ruiz, Jan Göpfert, Hans Christian Gils, Jann M. Weinand, Patrick Jochem, Jochen Linßen, Detlef Stolten

**Affiliations:** 1 Forschungszentrum Jülich GmbH, Institute of Energy and Climate Research – Jülich Systems Analysis, 52425 Jülich, Germany; 2 German Aerospace Center (DLR), Institute of Networked Energy Systems, 70563 Stuttgart, Germany; 3 RWTH Aachen University, Chair for Fuel Cells, Faculty of Mechanical Engineering, 52062 Aachen, Germany

**Keywords:** software coupling, data integration, scientific workflows, data models, transformation functions, metadata, reusability, interoperability, DataDesc Schema, ioProc workflow manager

## Abstract

In computational science, coupling research software and data into workflows is essential for addressing complex research questions and ensuring reproducibility. However, current metadata schemas rarely provide sufficient information about data models in software interfaces and datasets, hindering their effective integration into workflows. Additionally, most workflow management tools are not designed to handle metadata for process reproduction. This article refines the DataDesc metadata schema to annotate data models not only in software interfaces but also in datasets and presents an extension to the DataDesc framework for automatically comparing data models and identifying transformation requirements. The ioProc workflow manager is introduced to bundle shared transformation functions into adapter workflows and ensure transparent process documentation. Two use cases from energy systems analysis demonstrate the workflow design and implementation approaches. Overall, this article bridges the gap between abstract guidelines, such as the findability, accessibility, interoperability, and reusability (FAIR) principles, and researchers’ daily data- and software-driven analyses, promoting reusability, reproducibility, and transparency in science.

## Highlights

• DataDesc metadata schema describes data models in software and datasets
• Automated comparison identifies transformation needs for model and data integration
• ioProc adapter workflows bundle reusable transformations for flexible workflow design
• Approach enhances reproducibility, transparency, and FAIR-compliant research

## The bigger picture

In computational science, research increasingly relies on complex workflows that integrate diverse software tools and heterogeneous datasets. Despite this, the lack of standardized metadata describing data models in both software and datasets often hinders interoperability, reuse, and reproducibility. Researchers frequently spend significant time manually aligning and transforming data, which slows scientific progress and contributes to fragmented, non-reproducible workflows.

This work presents a metadata-driven approach to address these challenges. By refining the DataDesc schema to describe data models and extending it for automated comparison, researchers can systematically identify required data conversions. The ioProc workflow manager leverages this information to design reusable transformation functions and modular adapter workflows, ensuring transparent documentation and enabling reproducible, findability, accessibility, interoperability, and reusability (FAIR)-compliant analyses. This approach is broadly applicable across computational sciences, supporting the integration of diverse models and datasets. Looking forward, widespread adoption of metadata-rich workflows and public repositories of transformation functions could further accelerate research, facilitate automated workflow generation, and promote a more efficient and trustworthy scientific ecosystem.

## Teaser

This study refines DataDesc, a metadata-driven approach for coupling research software and data into reproducible workflows. Using automated data model comparison and the ioProc workflow manager to design reusable transformation functions, it identifies required data conversions and ensures transparent documentation, advancing interoperability and FAIR-compliant research across scientific domains.

## Introduction

### Motivation

In computational sciences and especially in energy systems analysis, the coupling of research software into comprehensive software workflows is a common approach for incorporating multiple perspectives into a scientific analysis and investigating complex research questions.^1^ In a workflow context, the sustainable handling of resources is particularly important in two aspects. On the one hand, these workflows represent in themselves flexible research tools. Reuse or adaptation of these workflows to changes in research questions in a fast, reliable, and low-effort approach can avoid considerable redundant development, implementation, and maintenance efforts but requires careful upfront design and additional effort.^2^ On the other hand, these workflows play a central role in scientific exchange, as they contribute to the transparency and reliability of scientific conclusions by enabling others to trace, interpret, and reproduce the results of these workflows.^3^^,^^4^ Workflow design therefore has the potential to positively increase the overall efficiency of a research community.

Usually, however, scientists are confronted with major challenges when creating and applying workflows that should be adaptable, reusable, and reproducible by others. Especially missing or difficult-to-process information about software and data resources represents a major obstacle. Particularly in a research field such as energy systems analysis, in which no uniform interface and data standards have been established and which is characterized by heterogeneous formats originating from different research disciplines, the provisioning of metadata, which includes information about individually used data models, is imperative.^5^ Another hindrance is the lack of computer-aided workflow design and building tools that cover and support both research software and data. In an environment full of initially incompatible data models, there is a strong need for reliable and robust data transformation and processing capabilities in order to combine input data and software models into a structured process. As long as there are no adequate tools supporting scientists in workflow integration or offering automation capabilities, data models must be manually researched and compared, which is a tedious and time-consuming task. In addition, transformation requirements must be identified manually and suitable transformation functions researched in a time-consuming process. A third challenge lies in the lack of traceability and reproducibility of workflows and workflow results. Even as there is no lack of workflow tools with which researchers can implement their model chains (see, for example, Kieser et al.^6^ or Mölder et al.^7^), the metadata accompanying these processes, which are relevant for their understanding and interpretation, must be recorded, linked, and stored separately, usually in a manual process. This additional effort, which has to be made for each individual model coupling, data processing, or transformation, is often not done consistently. This situation contributes to the current reproducibility crisis in science.^8^^,^^9^^,^^10^ An illustrative example is a study of Jupyter Notebooks, a common form of documenting scientific workflows and analysis, for their reproducibility. The study of Pimentel et al.^11^ showed issues with dependency documentation and execution of the notebooks and thus a lack of reproducibility, transparency, and adaptability.

Confronted with this situation, a cultural change is slowly taking place in energy systems analysis and other science communities, which places increasing importance on overcoming the associated challenges. In particular, the objectives formulated in the FAIR (findability, accessibility, interoperability, and reusability) principles are effectively promoting the adoption of metadata standards, as this directly contributes to all four principles.^12^ At the same time, the joint development and use of domain ontologies for the uniform description of software, data, and other research data artifacts, such as the Open Energy Ontology,^13^ is also gaining traction. As the progress of adopting the FAIR principles continues, numerous problems and uncertainties in the practical implementation of these guidelines are experienced in everyday science. To solve these, novel approaches and solutions are required with renewed urgency. As described by Leipzig et al.,^14^ there exists an explicit need for action in the area of metadata descriptions of data that are to be used as input by software models. The authors call for a common metadata standard that enables the description of file formats and data structures at the workflow level and their automated comparison in order to link individual elements of analytical processes. They claim that metadata tools are as important for practical and computational research as the software and data themselves. Leipzig et al.^14^ further show that despite the existence of numerous workflow tools, collecting and analyzing provenance, i.e., recording all activities that lead to the creation of a data object, is still a key challenge for workflow design.

The approaches presented in this article provide an answer to the question of how metadata-aware workflows, which cover software and data, can be implemented and documented to make a significant step toward the FAIR principles. In the related work section, current metadata schemas are first compared and examined with regard to their suitability for describing data models. Subsequently, an introduction to data comparison and workflow tools is given, focusing in particular on their capabilities for metadata processing in a scientific context. The methods section shows the extent to which the metadata elements of the DataDesc schema, which is designed to describe data models in software interfaces, are also suitable for describing data models in data files, and which structural refinements have been applied to facilitate this application case. Furthermore, an extension of the DataDesc framework for the automated comparison of metadata is presented, in which data models are compared, and any discrepancies are identified as transformation needs, whose machine-actionable descriptions can be used in the future for the automated identification and reuse of data transformation methods in modular workflow designs. In a second step, the ioProc workflow manager^15^ is presented, with which data transformation steps can be combined into so-called adapters as needed and sustainably reused in workflows. The individual process steps are also automatically documented as metadata. The results section presents two use cases from the context of energy systems analysis to demonstrate how DataDesc and ioProc can be used to design and implement transparent, reproducible, and well-documented scientific workflows. The discussion section, which emphasizes the main features of the approaches presented and their limitations, provides an insight into future work and closes this article.

This work hence presents an adaptable and extensible approach for improved design and reusability of scientific workflows incorporating software and data, rooted in and exploiting the benefits of metadata. Building upon prior work on the DataDesc schema, this paper introduces three key advancements: (1) refining DataDesc to describe dataset structures in addition to software interfaces, (2) extending the framework for automated data model comparison and transformation identification, and (3) integrating these capabilities into the ioProc workflow manager to demonstrate their practical application. The presented approach contributes to the FAIR principles and supports the transparency and reproducibility of workflows in a scientific community by adding automatic metadata generation.

### Related work

This section examines current metadata schemas with regard to their suitability for describing data models. Furthermore, different approaches for comparing data are contrasted. Finally, the role of workflow tools for computational sciences is established.

#### Metadata schemas

A metadata schema standardizes the description of artifacts within its scope by defining a set of metadata elements to be used.^16^ A metadata standard may further standardize the encoding format, allowed values, their representations, and so forth.^16^ Application profiles adapt subsets of one or multiple metadata schemas to tailor them to specific applications. They may further define custom elements, rules, best practices, etc. Many schemas, standards, and application profiles exist with different scopes. The scope refers to specific use cases that are supported but may be limited to, for example, a particular artifact type (such as images, software, datasets, etc.), scientific domain, or country.

Domain-agnostic standards that propose sets of elements to describe datasets include Dublin Core^17^; DataCite,^18^ which provides a mapping to Dublin Core; DCAT,^19^ which incorporates terms from Dublin Core as well as other controlled vocabularies; and schema.org’s dataset class^20^ that, in turn, is based on DCAT. None of these describes data models in detail. In addition, the general metadata of a dataset, such as *authors*, *titles*, *descriptions*, the content, and how to access it, are only defined in an abstract way using terms such as *encoding*, *file format*, *has part*, *about*, *variable measured*, *temporal or spatial resolution*, *and coverage*. Dublin Core and DCAT allow referencing a schema or standard using the property *conforms to*. However, there is no schema describing the data model provided.

RO-Crate^21^ and Data Package^22^ are approaches to bundling data and their metadata. For example, Data Package allows the specification of table schemas for individual files. OEMetadata^23^ is a domain-specific standard that builds on Data Package and encourages the referencing of concepts from controlled vocabularies and ontologies.

Representing *n*-dimensional datasets in the resource description framework (RDF), the RDF Data Cube vocabulary^24^ is not limited to tabular data. It represents the actual data themselves but uses the rich semantics of the RDF to describe information that would normally be part of metadata. D-REPR^25^ and the Software Description Ontology^26^ reuse the RDF Data Cube vocabulary to define the structure of a dataset. Other self-describing data formats include HDF5^27^ and NetCDF.^28^

More recently, Croissant^29^ and DataDesc^30^ proposed approaches that are independent of the dataset format, allowing for the referencing of terms from controlled vocabularies and the description of multidimensional and nested data structures. To increase the reusability of datasets within the machine learning community, Croissant supports the specification of data types and foreign key references. In addition, usage information, e.g., where the training, validation, and test splits are located, which columns are to be extracted, and how the values are to be parsed, facilitates the training of machine learning models. DataDesc is a domain-agnostic standard for describing software interfaces and their data models with metadata to facilitate model coupling, workflow composition, and software discovery, as well as integration in general. It builds on the OpenAPI specification, a widely used standard to document APIs, and provides a mapping to schema.org. DataDesc offers the possibility to describe multidimensional and nested function parameter structures and value ranges in runtime representations. Semantic concepts of variables, as well as their units or quantity types, can be described in a machine-actionable way by referring to terms from controlled vocabularies.

In its area of application for interface documentation, the DataDesc schema offers the highest level of detail and the greatest flexibility in the description of data models, enabling it to map variables and function parameters of any complexity. In addition, it integrates and references current metadata standards and can be used across domains and applications. However, there is currently no metadata schema that has been designed to map data models of datasets at a comparable level of detail, leaving a significant gap in automated support for the annotation and integration of datasets into complex software workflows.

#### Data comparison tools

To what extent and in what respect data are compared depends on the use case. We focus on the comparison of data models between datasets and software or service interfaces with the aim of integrating them into comprehensive software workflows. This requires consideration of both semantics and technical representations, including data structures, data formats, and value range specifications.

To compare different versions of files, utilities such as GNU Diffutils^31^ or Git’s diff^32^ highlight differences based on the line-based edit distances between them. With binary files, a comparison of lines or other text chunks is generally not meaningful; they are either classified as different or not.^31^ Specifying data models in separate metadata files circumvents this limitation.

To compare the contents of files beyond the purpose of comparing versions, the similarity of individual strings, such as the keys and values in a metadata file, can be calculated based on their token- or character-based edit distance (e.g., using the Levenshtein distance^33^). Often strings are normalized beforehand (e.g., by lowering and lemmatizing words). To also consider semantics, the vector similarity between strings can be calculated based on word embeddings (such as Word2Vec^34^ and GloVe^35^ embeddings). Alternatively, strings can be mapped to concepts in ontologies or lexical databases (such as WordNet^36^) and compared based on the semantic relations they define, such as synonyms or hyponyms. The meaning of a word or phrase depends on its context. Hence, consideration of the context is important. A-Match^37^ is an example of an application that uses both string metrics and ontologies to compare APIs. Following a human-in-the-loop approach, the system’s suggestions are visualized in a GUI and can be accepted or rejected.

When comparing more complex entities than strings, more information can be considered. For example, in addition to the parameter name, the allowed or actual value range can be considered (are the values within the expected range?), as well as the units (can they be converted into each other?). Consideration of the data structures is particularly important when comparing dataset and software interface descriptions. For RDF graphs, their structure, value ranges, and other constraints can be defined using the Shapes Constraint Language (SHACL).^38^ Corresponding validators can be used to validate RDF graphs based on these definitions. The MINT (model integration) framework^39^ also requires mapping datasets to the RDF. Variables are associated with a scientific variables ontology (SVO) concept. Based on the unified RDF representations, both content and structure are considered when matching a set of user-selected target variables to models that can output them.

When comparing data models between datasets and software or services, both semantics and data structures must be taken into account, ideally independent of the file format, to enable broad applicability. The functional extension of the DataDesc framework presented in this work encourages mapping to controlled vocabularies, is not restricted to a particular file format, and does not require the conversion of heterogeneous datasets into a unified representation, thereby addressing a key gap in existing approaches: the lack of automated, metadata-based support for identifying and resolving data-model mismatches between research data and software interfaces.

#### Workflow tools

Workflow tools play an important role in computational scientific work.^7^ The use of workflow tools ranges from high-performance computing environments to individual data analysis pipelines of scientists. Software solutions are correspondingly diverse, with software such as SLURM^40^ in HPC contexts not only managing workflows but also integrating resource management down to Jupyter Notebooks^41^ in individual data analysis pipelines with loose but easily adaptable workflows. The side benefit for the science of such workflow tools, especially when they provide a static workflow definition, is the creation of artifacts that are themselves suitable for documentation and the further creation of traceability and reproducibility for these workflows. A widely used workflow tool is Snakemake,^42^ which binds programs to flexible, statically declared workflows and provides a reproducible execution environment. An example of one of the more prominent applications of such tools, at least in energy systems analysis, is the coupling of models into complex workflows for multi-perspective scientific analysis.^43^

Most of the workflow tools used in the scientific community, Snakemake being a rare exception, were not originally developed specifically for scientific activities. As a result, few workflow tools incorporate scientific metadata processing and handling by design. The importance of scientific metadata is now being increasingly recognized.^44^^,^^45^ There, the tracking of data-related metadata and the computer-aided generation of metadata for generated datasets are central to the reusability and traceability of scientific results. The increasing popularity of the FAIR principles among both scientists^44^ and funding agencies^46^^,^^47^^,^^48^ only adds to the importance. The support of metadata information during workflows and of dedicated interfaces to scientific infrastructures, such as metadata hubs, is usually not natively covered by traditional generic workflow tools. Finally, workflow tools introduce their own complexity and require additional skills to be acquired by researchers. As workflow tool skills are a secondary concern in the scientific work environment, workflow managers with easy-to-learn principles and easy-to-use approaches are usually favored.

In summary, workflow tools often lack native support for scientific metadata and integration with scientific infrastructures, which makes data traceability and interoperability more difficult. Furthermore, their complexity and steep learning curve make them less attractive in scientific environments where ease of use is a priority.

In this work, we demonstrate, with our implementation of a scientific workflow in ioProc, both how lock-in effects with specific workflow managers can be avoided and how workflow tools can be designed so that scientists can apply them using their pre-existing knowledge of scripting and notebook environments.

## Methods

This section presents the developed methodological approach for integrating research software and data into modular adapter workflows. It builds on refinements and extensions of the DataDesc framework and introduces ioProc as a library-level workflow manager. The approach demonstrates how model workflows can be designed from reusable software components while ensuring reproducibility, transparent documentation, and meeting the specific needs of scientific research.

### Designing reusable workflows with DataDesc

**Figure 1:**
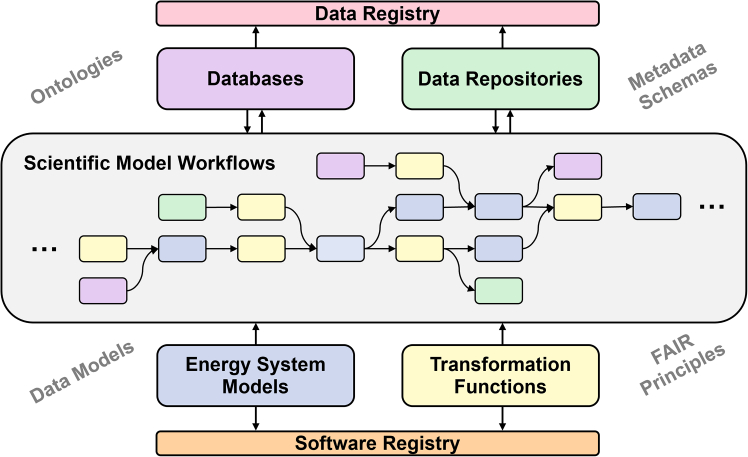
A modular workflow concept for the flexible integration of software and data in the context of a distributed research data infrastructure The original figure by Kuckertz et al.^49^ was supplemented by a software metadata registration component to facilitate the findability and reuse of energy systems models and data transformation functions.

This work takes up the modular workflow concept presented by Kuckertz et al.,^49^ which propagates a flexible approach to data and software integration and thus offers a lightweight alternative to the laborious introduction and adoption of static interface and data standards in energy systems research (cf. Figure 1). Along the workflow concept, information transfers between software modules via non-persistent in-memory data formats, such as integers, arrays, or classes, and is exchanged between software and data files based on persistent data formats, such as Excel, XML, or NetCDF4. This work addresses the integration of persistent data formats into software interfaces. To this end, the concept first requires the metadata description of software and data artifacts, whereby, in particular, their inherent data models are annotated in detail. Once these are available, the data models mapped by software interfaces can be compared with those used in data files in a second step. This provides information about the compatibility of data and interfaces and identifies transformation requirements that can be covered by data processing. Along this process, the concept aims at the reuse of software and data artifacts and their flexible integration into complex application-specific model workflows. In addition to the metadata descriptions, transformation functions form a central component, as they act as a link between the individual software interfaces and heterogeneously structured datasets within the domain of energy research. The concept intends for the transformation functions to be designed in a modular way, described transparently, and made available as open source so that they can be jointly used and sustainably developed within the research community. Overall, the concept is intended to improve the reuse of research software and the reproducibility and validation of study results, thereby promoting efficiency and academic exchange in the field of energy systems analysis.

In order to implement this concept in practice, metadata schemas are required that can be used to describe data models in detail, both in interfaces and in datasets. These must be compatible with each other insofar as the annotations created on their basis allow them to be compared exactly. In addition, the schemas should support semantic references to ontologies, such as the Open Energy Ontology^13^ developed for energy systems research, and machine-actionable exchange formats so that the comparisons can be carried out automatically. With DataDesc, Kuckertz et al.^30^ have already developed a framework that meets these requirements, at least for the formal description of software interfaces. DataDesc centers around a software metadata schema that describes the data models on which software interfaces are based. In addition, DataDesc effectively promotes the FAIRness of research software. As described in the related work section, there is currently no metadata schema for datasets that can be used to map their data models at a comparable level of detail. In order to present an approach to compensate for this omission, it is shown in the following to what extent the elements of the DataDesc metadata schema are also suitable for describing data models of datasets and how the schema structure has been refined for this purpose.

**Figure 2:**
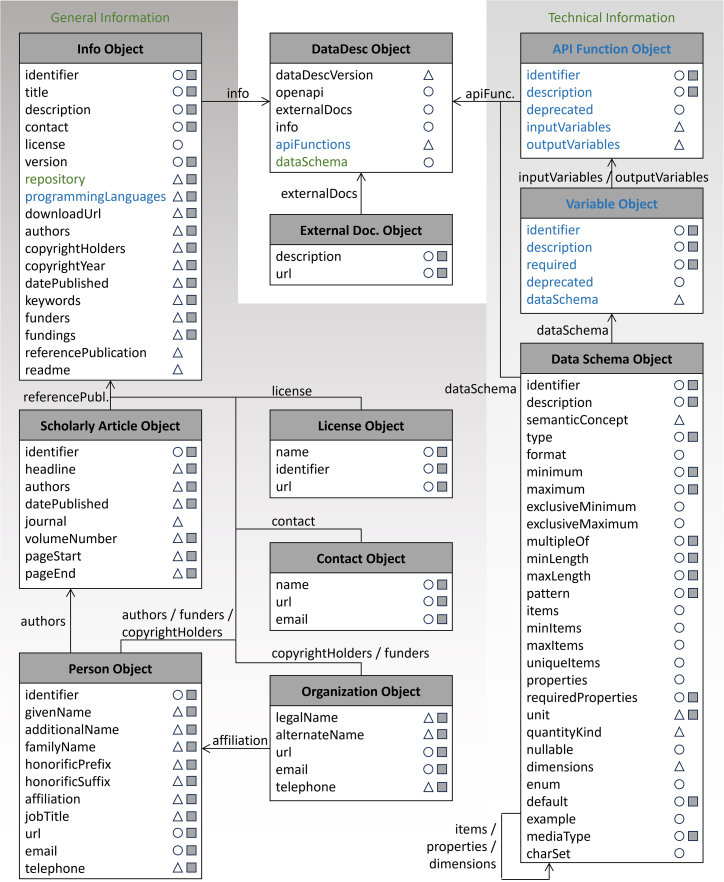
Structure and content of the DataDesc schema for describing software and their interface data models Both the general and technical information are organized in information objects, with arrows indicating the various relationships between them. DataDesc properties that map directly or via extensions with the OpenAPI specification are indicated by white circles and white triangles, respectively. Properties that map to the schema.org ontology are indicated by a gray square. The schema and figure from Kuckertz et al.^30^ have been slightly adapted, with the changes shown in green, to accommodate the description of data models mapped in files. Components written in blue are intended exclusively for the description of interfaces, while all other objects and properties are also used to describe datasets. Individual property definitions can be viewed at GitHub: https://github.com/FZJ-IEK3-VSA/DataDesc/blob/main/schema/DataDesc_schema_v1.2.md.^50^

The part of the DataDesc schema that is the focus of this work is the *data schema object* (cf. Figure 2). Its properties can be used to describe even deeply nested data models in terms of contents, formats, value ranges, and structures. The contents of data structures can be documented using *description* and clearly assigned to individual ontology classes using *semanticConcept*. It is of no consequence whether these contents are expected by a software interface or provided by a data file. The properties *type*, *format*, *mediaType*, and *charSet* can be used to describe data model components of both persistent and non-persistent variables. For example, the type and format can be used to specify not only the valid file type for variables that expect a file as input but also the assumed data structure within a transferred file. The various *minimum* and *maximum* properties can be used to define value ranges that must be adhered to when using an interface in order to ensure error-free data processing. With regard to files, these properties describe specific value ranges contained in the dataset. Finally, the description of nested, grouping, or dimensionally resolving data structures with *items* and *properties* remains the same, regardless of whether they are used in a function variable of complex type or a data file. Overall, it becomes evident that the data schema object can be used to describe data models in files in the same way as in software interfaces.

In Figure 2, object types and properties of the DataDesc schema that are intended exclusively for the description of interfaces are highlighted in color. It can clearly be seen that the majority, including the part required for the description of data models, is just as suitable for the annotation of data files as it is for software interfaces. The only structural difference is that the data schema object is assigned directly to the *DataDesc object* when describing files. By reusing the schema, the exchange format of the DataDesc framework can also be reused, which inherently ensures the homogeneity of the metadata descriptions.

On this basis, the DataDesc framework has been expanded to include analysis functionality that enables the direct and systematic comparison of a data model mapped in a file with the one implemented in a software interface and reveals the degree of their compatibility. In addition to the structures of the respective data models, the properties of the variables they contain, such as dimensionality, value ranges, and units, are also contrasted individually. During the comparison, the software interface serves as the reference point against which the dataset’s data model is assessed for compliance with the required interface structures. While the absence of expected variables or deviations from expected units or value ranges reduces the degree of compatibility, additional information contained in the file that is not required by the interface is ignored and does not have a negative effect. Essentially, the comparison process checks whether the information required by the software interface is available as a subset in the data file.

As a result of a comparison, a report is generated that provides detailed information about (in)compatibilities. Starting from an overall result that reflects whether the comparison has revealed discrepancies or not, the report goes into more and more detail along the hierarchy of the data model until it becomes clear at which points in the data model the requirements have not been met. This accurate error indication is intended to support researchers in the design and reuse of computational workflows, to avoid errors in data processing, and to make software and data integration more efficient. At the same time, reports follow the DataDesc structure so that they can formally represent compatibility information as independent, machine-actionable DataDesc documents and can be further processed automatically.

If a file is to be used as input by a software despite incompatibilities, transformation requirements arise that must be covered by data processing. Therefore, necessary transformations can vary considerably in their complexity and scope, ranging from simple unit conversions to extensive data restructuring. As the report provides information about the type of incompatibility, it can be used to identify suitable transformation functions. For example, differences in currency formats can be resolved with Currency Conversion for Python (CuCoPy),^51^ while discrepancies in the spatial or temporal resolution of the data can be addressed with Spagat^52^ or tsam.^53^^,^^54^

Many such transformation functions are currently available as open source. However, even if they are registered in software catalogs, as indicated in Figure 1, they cannot yet be automatically suggested or selected on the basis of identified transformation requirements. With their formalized information on data models and incompatibilities, the machine-actionable DataDesc reports form a technical basis for the goal-oriented findability and reusability of data processing software.

Once the model and data components required for an analysis have been connected using the transformation functions identified via DataDesc, the modular concept subsequently requires them to be combined into transparent and reproducible computational workflows.

### Implementing reproducible workflows with ioProc

ioProc is a library-level workflow manager, which distinguishes it from other tools such as the popular Snakemake workflow management system.^7^ Snakemake operates on the software level, so it ties individual software pieces together into a larger workflow. ioProc, on the other hand, chains functions, called actions in this context, into a reproducible workflow specified by a static definition file, called an adapter. This adapter also serves as part of the scientific documentation of the workflow and can be used to run the workflow repeatedly and reproduce analysis results. In addition to the workflow itself, ioProc also writes log files, with one dedicated only to data modifications to adhere to good scientific practices. All read and write operations to and from data structures inside of ioProc actions are thus documented and traceable for each execution of the workflow. Furthermore, ioProc follows the open-closed principle; thus, it is open to extensions but closed to modifications. This manifests in different aspects of the software. For example, all actions are saved in an action folder, including the default set of built-in actions, which ioProc can generate on request. The ioProc-generated actions are standardized, but as they are stored as a module, accessible by the user and outside of the ioProc software, this opens them up for modification and extension without needing to modify ioProc itself. The user can furthermore declare their own actions in additional files. ioProc, if configured with the location of these extension files, can pare these actions and apply them in a user-specified workflow. In addition, users can share their actions with others so that they can use them in their own workflows. To improve reusability, files with ioProc-compatible actions can be modified with little mockup code to make them independent from an ioProc installation. It is then possible to use these actions like ordinary Python functions in environments without ioProc, such as a framework, a Jupyter Notebook, or another workflow tool. ioProc thus creates an environment that is geared toward good scientific practices with a unique focus on library-level workflows.

## Results

**Figure 3:**
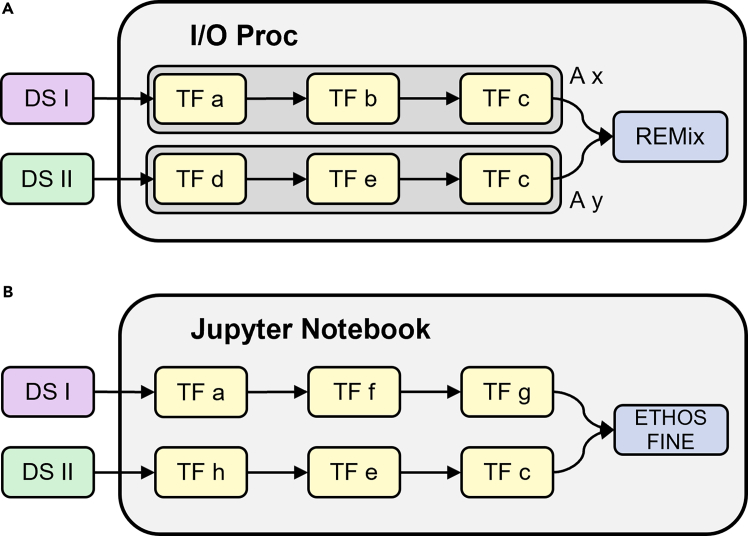
Workflow comparison of ioProc and Jupyter Notebook Schematic comparison of an ioProc (A) and a Jupyter Notebook software workflow (B) that use the same datasets (DSs) as input and transfer their contents into the energy system models REMix and ETHOS.FINE using individually combined transformation functions (TFs) and adapters (As).

To illustrate our design and implementation of reproducible software workflows from reusable software and data components and to show the general applicability of the approaches presented, two independent and simplified model workflows are compared in this section (see Figure 3). With REMix (renewable energy mix)^55^ and ETHOS.FINE (Framework for Integrated Energy Assessment [FINE] of the Energy Transformation Pathway Optimization Suite [ETHOS]),^56^^,^^57^ two mathematical optimization frameworks with a similar scope from the field of energy systems research are employed. Both application cases are based on pre-existing models that are instantiated from the two frameworks. While both models contain individual sets of predefined parameters and data, they also share some input datasets. The shared input data include unresolved data regarding energy technologies on the one hand and temporally and spatially resolved renewable energy potentials on the other. During data processing within the two workflows, these differently structured datasets are adapted to the data models expected by the frameworks’ interfaces along individual transformation steps, with some of the modular transformation methods reused in both workflows. While the REMix workflow is implemented using ioProc and maps a file-based import of the data, the ETHOS.FINE workflow is implemented using Jupyter Notebook on the basis of a script-based data import.

When selecting the application cases, the focus was on the fact that they differ in a number of relevant characteristics but remain structurally comparable. The model calculations carried out are realistic in terms of data structures, formats, contents, and value ranges. Beyond that, however, they do not represent conclusive analyses of energy systems.

### Shared input datasets

Both workflows ingest technology catalogs from the Danish Energy Agency,^58^ which provide a comprehensive but unresolved dataset of techno-economic data for technologies commonly used in energy systems analysis. The data are provided under the CC BY 4.0 license in Excel spreadsheets grouped by technology type, such as electricity and heat generation,^59^ storage,^60^ and renewable fuel production.^61^ Each Excel file contains the worksheet *alldata_flat*, in which all data points have been converted into a row-by-row format and can therefore be easily processed by machines.

In addition, in the application cases, both workflows use photovoltaics (PV) and wind generation time series from Pfenninger and Staffell.^62^ The time series are geographically and temporally detailed at the country level, in this case for Germany, and in an hourly resolution for all weather years between 1980 and 2019. The data are provided by the website *renewables.ninja*^63^ under the open CC BY-NC 4.0 license as CSV files, which are structured into two columns: timestamp and normalized generation.

**Figure 4:**
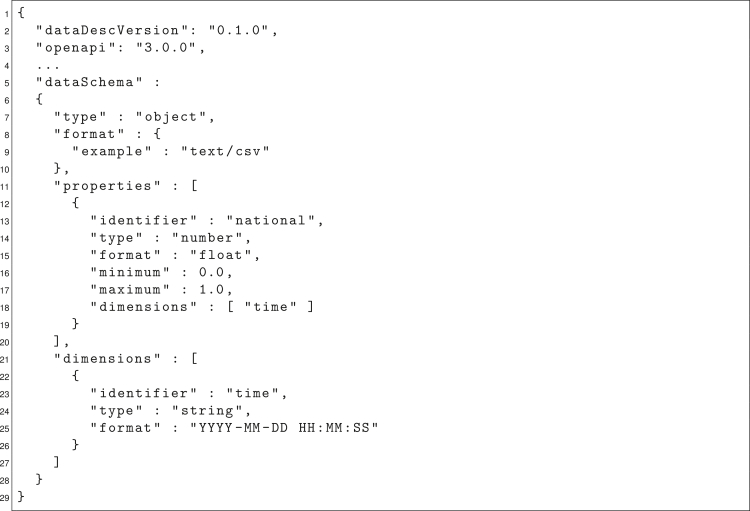
DataDesc document describing a PV generation data model Abridged DataDesc document describing the data model of a country’s PV generation time series in an hourly resolution as provided by *renewables.ninja*.^63^

Figure 4 shows an abridged DataDesc document describing the data model of the PV generation time series for Germany. The CSV file comprises a tabular structure consisting of two columns: *national* (lines 13–18) and *time* (lines 23–25). While *national* holds the capacity factor float values for Germany in the range between 0 and 1, *time* represents the temporal dimension, indexing the values using the coordinated universal time (UTC) format.

### Shared transformation functions

In both workflows, the same fundamental micro-transformations of data need to be performed. These operations were implemented to be compatible with ioProc, and the general ones were applied in both the REMix and ETHOS.FINE workflows. Both workflows share the following transformation steps (we denote the exact function name of the ioProc action in brackets for each step).

The workflow starts with reading the source data into a memory data structure for further processing (action *read_excel()*). We then clean the input data from whitespaces and special characters in the text-based raw data (action *strip_data()*) and convert numerical data to Python numerical types (action *remove_non_digit_values()*). The next steps are the reduction of the dataset to the technologies we are interested in (in this case, gas turbines, Li-ion batteries, heat pumps, and electrolyzers) (action *filter_technologies()*) to the relevant technology parameters (action *filter_parameters()*) and finally to the target years (action *filter_years()*) and the mean estimate (action *filter_estimate()*). Finally, we exclude all columns with irrelevant data for our use case from the dataset (action *drop_columns())*.

In the next phase of the data preparation, we have to make adjustments to the dataset labels, for example, to be compatible with the REMix conventions. We hence rename the technologies (action *rename_technologies()*) and parameters (action *rename_parameters()*) to their given standard names. Afterward, we convert all values to consistent base units (action *convert_units()*) and discount the costs to a uniform reference year (action *convert_currency()*). For the cost conversion, we delegate the task to the CuCoPy library,^51^^,^^64^ which we call from inside the action. CuCoPy is an open-source software package developed to streamline the integration of heterogeneous currency data into research software. The package enables the conversion of currencies from over 260 countries, and it has the ability to adjust financial data for inflation dating back to 1960 on an annual basis. As CuCoPy is independently released under the open MIT license, it seamlessly integrates into existing applications but also works autonomously within processing workflows like the ones described here.

### REMix workflow utilizing ioProc

REMix is a modular codebase that allows setting up linear optimization models and was developed by the Institute of Networked Energy Systems (VE) at the German Aerospace Centre (DLR) to address research questions in the field of energy systems modeling.^55^ The framework is programmed in Python and relies on GAMS as its mathematical programming language. REMix can be used to address problems related to the optimal design and operation of future integrated energy systems, both for target systems and transformation pathways. It implements methodologies such as Pareto optimal search, modeling to generate alternatives, perfect foresight, and myopic optimization. One of the most important strengths of REMix is its high-performance scalability, as it is designed for processing large spatiotemporal optimization scopes. This makes it feasible to conduct path optimizations of high complexity with multiple years in hourly resolution and a high spatial resolution, including balance area sizes up to the continental level and individual transmission lines. REMix is based on an implementation-agnostic data model, which makes it applicable for different scopes. The data model consists of five basic building blocks, nodes, and goods. Nodes represent installations or connection points, and goods describe everything that is exchanged within the system. Converters balance the consumption and production of goods, storages allow the retention of goods across time, and transfer links allow the transfer of these goods between nodes. Sources and sinks allow the input and output of goods from and into the modeling scope, respectively. Complemented by indicators, users can model complex accounting relations, such as carbon taxes or electricity market prices.

The primary input to REMix models consists of structured data, stored in CSV or DAT files, which are loaded into the REMix model at the beginning of each model execution. These datasets are comprehensive, covering all aspects of the energy systems model being analyzed. They also play a crucial role in the configuration of the model, as REMix is a data-driven framework. Therefore, the combination of a specific REMix software version and a complete dataset constitutes a REMix instance, which is reproducible. In most cases, scientists do not create entirely new datasets from scratch, since research questions rarely focus on completely novel or previously not modeled systems. Instead, most modeling work is derived from a basic energy system model that defines the scope and area of interest. This requires scientists to provide data for specific parts of the model, such as particular technologies or regions, and properly link them to the overarching energy system data. Technically, this often involves modifying or replacing certain parts of an existing dataset. The core dataset that represents the energy system is referred to as the baseline dataset. For a dataset to be usable as a baseline, it must conform to the REMix conventions and be complete enough to execute the model without the need for additional data.

This application case focuses on the common task of adding specific data to a baseline model in REMix. To this end, we developed an exemplary workflow that integrates publicly available open data into a baseline dataset. The main tasks involved are transforming the data to fit REMix conventions and creating a new set of REMix-compatible input data files. We implemented this workflow in ioProc, which consists of a series of distinct, specific actions. The implementation is independent of any particular workflow or model context, enabling the exchange and sharing of source code between the REMix and ETHOS.FINE workflows.

The REMix workflow begins with 12 general data cleaning and transformation actions that focus on parsing and interpreting the input data format. These general actions are followed by REMix-specific steps to clean and transform the raw input data to comply with REMix conventions. In the final stage, the data are converted into the standard input data structure for REMix: a two-dimensional table format with standardized labels and columns. The next step involves incorporating the new dataset into the baseline model. To do this, we read the baseline dataset, parameterize the entire model instance in REMix, and replace the relevant subset of the existing baseline data with the new, more detailed dataset. In this example, the new dataset introduces additional technologies, which must also be declared in REMix. This step completes the data preparation process. At this point, the first dataset is ready to be written as REMix input files, marking the final step of the workflow.

In this example, we incorporate two datasets, with the second being time-series data. The time-series data are handled using the same workflow described above, though the required transformations are fewer since the data are already in a format compatible with REMix. As a result, we can skip the cleaning and conversion steps. The next steps involve reducing the dataset to the target year for our model run and converting the labels to the REMix standard. The data are now ready to be integrated into the base REMix model, following the same procedure as for the technology dataset. We have now created a complete REMix input dataset consisting of the baseline dataset and the incorporated technology and time-series data. These files are now ready for processing by REMix.

Reading a dataset into REMix is facilitated by a call to the *read_remix_csv* function, which we have documented as part of this project with the DataDesc schema to provide additional meta information and make it machine readable. The description includes a detailed description of the two input parameters *file* and *schema,* which were described as separate variables with their own data schemas. While *file* is a simple string that refers to a CSV file, *schema* is a complex data structure that refers to a JSON file that is composed of various fields (including *name*, *title*, *type*, *isAbout*, etc.). The return value of the function is the REMix interface internal data format, which is a *pandas.DataFrame*.

To demonstrate how specific operations in one workflow can be generalized, such that they become applicable in another workflow, we implemented two operations in the action format of ioProc and used them in the ETHOS.FINE workflow as well. The selected actions are the reading of the raw source data into memory (more precisely, into *pandas.DataFrames*) and the currency conversion, which are based on CuCoPy. This demonstrates that atomic transformation operations can be used across different workflow implementations and to create a shared basis of operations.

The full ioProc workflow encompasses the raw input datasets and the baseline REMix model data, the workflow specification in the ioProc YAML file format, and the action folder containing all ioProc actions. This bundle is published alongside this publication on GitHub and constitutes a reproducible workflow artifact.^65^^,^^66^ With the documented ioProc version, it is now possible to reproduce the workflow. Furthermore, in the spirit of good scientific practice, the action folder, containing all operations needed for the workflow, is included and describes all scientifically relevant transformations of the data. It therefore constitutes a different form of documentation, which can be used to examine the process in the future, independent of an ioProc installation.

This approach supports reproducibility and transparency, as a separation of the basic data, the workflow software components, and the scientifically relevant transformations is achieved. The ioProc actions can be used in the future to rebuild the workflow in any Python context, since they do not depend on an ioProc installation. But also the reverse is true. Using an ioProc action in a script is just as easy as modifying existing source code in notebooks or scripts so that it can be used as an ioProc action. This enables code reuse without compromising the ability to continue using the same code in existing scripts. The transformation into ioProc actions comes with the added benefit that the metadata handling, logging, and basic change tracking of ioProc workflows are applicable without additional work.

From a research software engineering perspective, the ability to use ioProc actions in other environments is an enabling feature, as the individual testing of actions becomes easy to implement. Such tests also serve as another form of scientific documentation beyond their usual benefit of guaranteeing technical correctness. ioProc provides scientists a framework in which to build flexible and granular transformation operations, which can be combined and extended as needed and applied in a broad range of software environments, reaching from scripts and notebooks to full software applications.

In summary, the REMix workflow demonstrates how ioProc enables modular, reusable, and transparent data transformations. Preprocessing steps such as raw data reading, format conversion, and currency normalization are encapsulated as independent actions, which improves clarity and makes the workflow easier to reproduce. The integrated metadata handling, logging, and limited version tracking further support transparency and reliable reuse of the workflow. Without ioProc, these preprocessing steps would need to be performed with *ad hoc* scripts or manual procedures, which are more error prone, harder to document, and difficult to reproduce.

### ETHOS.FINE workflow utilizing Jupyter Notebook

The FINE is part of the ETHOS at the division Jülich Systems Analysis of the Institute of Energy and Climate Research at Forschungszentrum Jülich.^67^ The Python-based framework serves as the basis for a diverse set of energy systems analyses conducted at the institute.^68^^,^^69^^,^^70^^,^^71^^,^^72^ Their outcomes support investment decisions and policy-making in the transformation toward fully renewable energy systems. ETHOS.FINE leverages the capabilities of the general framework for linear optimization, Pyomo, to model linear optimization problems for energy systems analysis. ETHOS.FINE introduces the concepts of source, sink, storage, and transmission components at different spatial and temporal resolutions as building blocks of an energy systems model. Typical problems that can be analyzed with such models are cost-optimized future energy systems of single buildings,^68^ municipalities,^69^^,^^70^ countries,^71^^,^^72^^,^^73^ or worldwide energy carrier transport.^74^ Furthermore, ETHOS.FINE incorporates methods for spatial and temporal aggregation,^53^ tackling the problem of long calculation times for large-scale mixed-integer linear energy system optimization problems.

The relevant model information is stored in the *EnergySystemModel* container class. This class holds general information such as units and location lists, as well as instructions for solving the algorithm. In addition, instances of components are added to the *EnergySystemModel* class in a modular way with the help of the *EnergySystemModel.add()* method. The output of the optimization consists of values for the optimal design and operation of a minimum-cost system.

For the documentation of the programmatic interface, the constructors of the *EnergySystemModel* class and the component classes were described using DataDesc (*FINE.json* in the supporting material).^65^^,^^66^ Lines 1–243 contain general metadata of the software. From line 264 onward, the *EnergySystemModel.add()* constructor function is described. The data schema description includes an explanation of the properties, types, ranges, and default values and indicates required properties. Based on an example from the ETHOS.FINE repository,^56^ two Jupyter Notebooks have been created that contain the processing of raw input data to the format that is needed for the model (*01_data_processing.ipynb*) and for model generation (*02_model_calculation.ipynb* in the supporting material).^65^^,^^66^ The preprocessing step reads time series for renewable energy potentials and energy demands from existing CSV files, as well as techno-economic parameters from a JSON file. The renewable energy potential time series collected from the *renewables.ninja* website can be inserted into the model’s input CSV without transformation because they are unitless values between 0 and 1. Parameter values for an absorption heat pump have been collected from the dataset of the Danish Energy Agency. Here, the preprocessing methods described in the previous sections have been used to read and transform the data. The raw data have been read from Excel into a dictionary format with the methods described in the section shared transformation functions. Economic values are described as EUR/MW in the input data while the model uses EUR/GW. The transformation has been conducted with the *convert_units()* method. Also, all monetary values are transformed to the year 2022 using CuCoPy.

In contrast to REMix, where one of the core functions with very few parameters was described, only a small function was described for ETHOS.FINE: the *EnergySystemModel.add()* function. The *add()* function expects many more parameters, some of which have custom complex data types that are not found in the standard Python package library. These user-defined data type descriptions can be summarized in unique schemas and referenced multiple times in the document to avoid redundancy.

**Figure 5:**
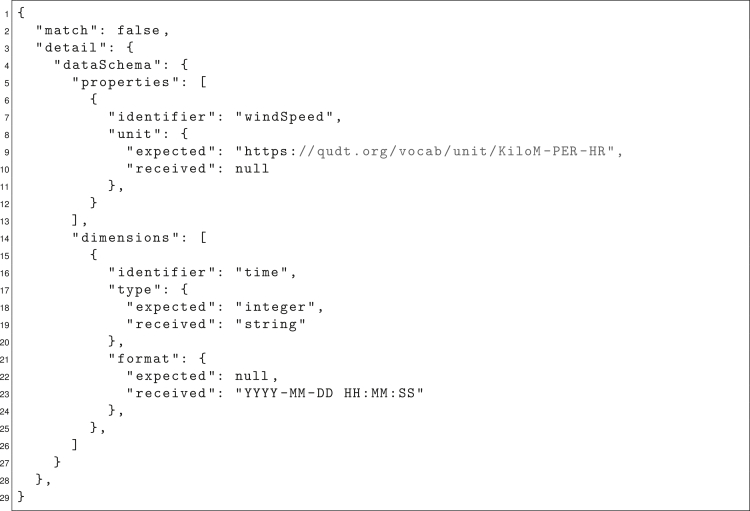
DataDesc report comparing software interface and wind generation data Abridged DataDesc report^65^^,^^66^ contrasting and comparing individual data model components of the ETHOS.FINE software interface and the wind generation time series from Pfenninger and Staffell^62^ as provided by *renewables.ninja*.^63^

A helper script (*tools/comparison/main.py*) was used to automatically identify transformation needs between the API and the datasets. The script was called via the command line, where a DataDesc interface description, an input file description, and a file path for the output file were specified. As software descriptions usually consist of more than just one interface, it was also necessary to specify which function and which parameter of said function should be compared with the input data. The comparison is always carried out from the perspective of the interface, meaning that a comparison is only successful if the input data serve all the required parameters of the interface. On execution, JSON-formatted report files were generated (see the example in Figure 5), which provide information about overall compatibility (line 2) but also more detailed information about missing expected data (lines 9–10) and mismatching data formats (lines 18–23).

For ETHOS.FINE, the workflow demonstrates how automated interface validation and metadata documentation ensure accurate and consistent input data. Without this approach, each dataset would require manual verification, increasing the risk of errors and reducing reusability. With this workflow, transformations are systematically checked against the software interface using DataDesc’s schemas and helper scripts, producing reports on missing or incompatible parameters while ensuring reproducibility, transparency, and easy reuse of preprocessing methods in other projects or environments.

## Discussion

The coupling of research software and data into comprehensive workflows is a common procedure in the computational sciences to help answer complex research questions. Therefore, workflows are not only relevant as versatile and reusable software tools but are also a central component of scientific exchange when it comes to tracing, reproducing, and interpreting results data and, ultimately, increasing the reliability of derived findings.^3^

Leipzig et al.^14^ point out that metadata play a special role in the context of reproducible computational research. They characterize the description of input or raw data and file intermediates as they are processed in workflows as an essential core task of metadata. However, since none of the established metadata schemas provide a sufficient description of the data models used in datasets and software interfaces, information that can be automatically processed to enable easy coupling of components in workflows is rarely available. The presented refinement of the DataDesc schema, which allows data models to be annotated not only in software interfaces but also in datasets, closes this gap and thus represents a necessary addition to established domain-agnostic metadata standards, such as Dublin Core or schema.org.

The added value resulting from the ability to formally describe information of this kind in the form of metadata is manifested in the downstream applications that this enables. Leipzig et al.^14^ mention file format and content sanity checks, which are defined by input metadata but implemented at the workflow level, as a prominent use case that has not yet been realized. To address this use case, the DataDesc framework was expanded to include a tool that automatically compares not only data content and formats but also data structures and value ranges, identifies discrepancies as transformation requirements for successful integration, and describes them in a machine-actionable form. The ioProc workflow manager presented here then offers the option of addressing identified transformation requirements by bundling and integrating transformation paths in the form of modular adapters and making these available for reuse. Based on the utilized modular workflow concept for the integration of software and data components, DataDesc and ioProc support researchers in their manual design of research software workflows.

Furthermore, the DataDesc schema refinements and the ioProc workflow manager are not limited to the presented implementation. Since DataDesc is designed as a domain-agnostic and tool-independent metadata schema, it can be integrated into other workflow environments. The generated metadata can be exchanged through standard interfaces or common serialization formats (e.g., JSON-LD), allowing interoperability between different workflow systems, including ioProc and Snakemake.

A promising application based on this comes from the innovative field of computer-aided workflow design and integration, which could use such machine-actionable information to automatically identify suitable data conversion and integration paths for model chains and suggest them to researchers. The web-based scientific data processing platform Galaxy,^75^ for example, serves as a platform for describing, sharing, and publishing scientific calculation processes and is designed to facilitate their discoverability and reusability in an accessible and interoperable manner. Kumar et al.^76^ have developed an approach that enables the platform to suggest tool combinations based on usage patterns identified via deep learning. At this point, DataDesc’s automated comparison of the data models would provide information about transformation requirements and a qualitative assessment of the proposed workflows. Computational sciences have access to huge amounts of heterogeneous research data and research software. These can no longer be fully evaluated using conventional data and tool searches, which often leads to low reuse rates and redundant developments.^77^^,^^78^ Against this background, the computer-assisted selection and integration of components into one’s own research workflows is of particular importance.

In addition to designing and implementing scientific software workflows, ensuring that they are used transparently and comprehensibly in studies and analyses remains a key challenge. In this context, the problem of reproducibility in research has already been comprehensively documented.^79^ Leipzig et al.^14^ describe computational process pipelines as a widely used method for performing scientific analyses, which encourages parameterization and configuration that promote reproducibility. With ioProc, a lightweight workflow manager has been released that natively implements the modular workflow concept for integrating research data and software in the form of adapters within reproducible and reusable data pipelines.

Overall, this work advances reproducible computational research by refining the DataDesc schema to formally annotate data models, enabling automated comparison and detection of transformation requirements, and implementing these capabilities through the ioProc workflow manager. Together, these developments bridge the gap between abstract guidelines, such as those systematized in the FAIR principles, and the practical realities of research workflows, balancing the effort of metadata documentation with tangible benefits in the design, execution, and reproducibility of complex scientific analyses.

### Limitations

In this work, we have for the first time presented application examples to demonstrate in a coherent and comprehensive manner how metadata-based design and modular coupling processes interact and can be implemented. Although the FAIR principles and open-source and open data are already becoming increasingly widespread, it will take some time before the publication of entire workflows and modular transformation functions becomes standard practice in science. As the approach presented here benefits from the high availability of existing and well-annotated software and data components to quickly identify and integrate needed components into the user’s own workflows, its effect is initially limited and will only develop its full potential with the increasing expansion and use of digital research infrastructures such as workflow repositories and software registries. The DataDesc schema and the ioProc workflow manager are complementary components in this context, which, through further integration into the infrastructure, will offer even better embedded and holistic solutions for everyday use.

The approaches and tools presented in this paper aim at loosely coupling models via transformation adapters, with the goal of achieving flexible interoperability between calculation steps that may differ in data formats, data types, syntax, systems, and execution times. No adaptation of datasets or software to predefined data and interface standards is required. In application contexts where workflows are mostly hard coupled, the direct benefit of the presented approach is limited. In co-simulation, for example, models are usually provided with standardized interfaces that do not require further transformation when interconnected. Only when hard and loose coupling of models is combined within hierarchical workflows should clearly annotated and reusable adapters be used again.

### Outlook

Based on this work, future studies could further develop both the presented implementation and design aspects. A reasonable next step would be the automated annotation of data files, starting with a selection of especially compatible formats and working from there. Also, further metadata publication pipelines should be included in the framework in order to supplement existing connections to software publication platforms with those for data publications, such as the Open Energy Databus.^80^ Correspondingly, the creation of a public transformation function repository or catalog with a stable API together would greatly improve transparency and contribute to findability and reusability, i.e., core values of the FAIR principles. Based on such data and transformation function availability, tools can be developed that automatically identify the needed transformation functions for connecting two different data formats (such as the format of a data source and the format of a model data input) and the ability to create lists of needed and missing transformations from which scientists build their workflows. This would then open up the possibility to further investigate limitations and best practices for automated generation of workflows in different scientific contexts. Additional tooling can then be developed, such as recommendation systems for transformation functions or alternative workflow formulations based on external factors such as license compatibility. A different direction for future work would be the improvement of the workflow tools themselves, such that they include automatic tracing of data modifications along a specified workflow and include this information in metadata formats. This can then be expanded toward systems that also trace the reason behind modifications and support scientists in adding contextual information during their workflows in a user-friendly way.

## Resource availability

### Lead contact

Further information and requests for resources on the DataDesc framework should be directed to and will be fulfilled by Patrick Kuckertz (p.kuckertz@fz-juelich.de).

Further information and requests for resources on the ioProc workflow manager should be directed to and will be fulfilled by Benjamin Fuchs (benjamin.fuchs@dlr.de).

### Materials availability

This study did not generate new unique reagents.

### Data and code availability

• The DataDesc metadata schema and the source code of the developed comparison tool for DataDesc documents are publicly available under the open MIT license at https://github.com/FZJ-IEK3-VSA/DataDesc.^50^ In addition, these resources have been made available in their current v.1.0 in the JülichDATA repository under the CC0 public domain dedication at https://doi.org/10.26165/JUELICH-DATA/DLCYV5.
• The ioProc workflow manager in v.2.2.0 under the MIT license was used for this work and is publicly available at https://pypi.org/project/ioproc/ and as a Git repository at https://gitlab.com/dlr-ve/esy/ioproc. Furthermore, the add-on ioprocmeta was also used in this work and is available under the BSD-3-clause license at https://pypi.org/project/ioprocmeta/ and as a Git repository at https://gitlab.com/dlr-ve/esy/ioprocmeta.
• The example and documentation files for the presented application cases are available in the JülichDATA repository https://doi.org/10.26165/JUELICH-DATA/VLBZIA^66^ and in the corresponding Git repository https://github.com/dlr-ve-esy/modelcouplingworkflows.^65^ Both are published under the BSD-3-Clause and CC-BY 4.0 licenses.

## Acknowledgments

The authors would like to thank the Federal Ministry for Economic Affairs and Climate Action of Germany (BMWK) for supporting this work with a grant for the project LOD-GEOSS (03EI1005A-G). Furthermore, the authors are grateful to the German federal government, the German state governments, and the Joint Science Conference (GWK) for their funding and support as part of the NFDI4Ing consortium, managed by the German Research Foundation (DFG) – 442146713. This work was also supported by the Helmholtz Association as part of the program “Energy System Design.”

## Author contributions

Conceptualization, P.K. and B.F.; methodology, P.K., B.F., and J.S.; software, B.F., H.G., K.K., and J.S.; validation, K.K., H.G., J.S., B.F., and P.K.; investigation, P.K., B.F., J.S., and J.G.; data curation, H.G., B.F., E.A.R., and J.S.; writing – original draft, P.K., B.F., J.G., E.A.R., K.K., H.G., and J.S.; writing – review & editing, P.K., B.F., J.S., H.G., K.K., E.A.R., J.G., H.C.G., J.M.W., P.J., and J.L.; visualization, P.K.; supervision, P.K., B.F., J.M.W., J.L., and P.J.; project administration, P.K., B.F., J.L., and P.J.; funding acquisition, P.J., and D.S.

## Declaration of interests

The authors declare no competing interests.

## Declaration of generative AI and AI-assisted technologies in the writing process

During the preparation of this work, the authors used the tools DeepL and ChatGPT to check grammar and spelling in a few places and to make minor improvements to readability and style. After using these tools, the authors reviewed and edited the content as needed and take full responsibility for the content of the publication.
